# Supplementary material for: Empirical methods for controlling false positives and estimating confidence in ChIP-Seq peaks
Source: BMC Bioinformatics. 2008 Dec 5;9:523. doi: 10.1186/1471-2105-9-523 (PMC2628906; doi:10.1186/1471-2105-9-523)
Supplement: Additional File 1 — A variety of html documents from the USeq web site detailing the available applications, their best usage, output file type descriptions, command line menus, etc. [file 1471-2105-9-523-S1.zip › USeqUserGuides/JavaDoc.zip!/JavaDoc/index-files/index-22.html]

W-Index


|  |  |  |
| --- | --- | --- |
| **Overview** \| Package \| Class \| Use \| **Tree** \| **Deprecated** \| **Index** \| **Help** | |  |
| **PREV LETTER**   **NEXT LETTER** | **FRAMES**    **NO FRAMES**     **All Classes** |  |


A B C D E F G H I J L M N O P Q R S T U V W X Z 

---


## **W**

**Wig2Bar** - Class in trans.misc: **Wig2Bar(String[])** - Constructor for class trans.misc.Wig2Bar: **WilcoxonRankSumTest** - Class in trans.main: Implementation of a Wilcoxon Rank Sum Test. **WilcoxonRankSumTest()** - Constructor for class trans.main.WilcoxonRankSumTest: **WilcoxonSample** - Class in trans.main: Used by the `WilcoxonRankSumTest`. **WilcoxonSample(float, boolean)** - Constructor for class trans.main.WilcoxonSample: **WilcoxonSamplePair** - Class in trans.main: Used by the `WilcoxonSignedRankTest`. **WilcoxonSamplePair(float, float)** - Constructor for class trans.main.WilcoxonSamplePair: **WilcoxonSamplePair(float)** - Constructor for class trans.main.WilcoxonSamplePair: **WilcoxonSignedRankTest** - Class in trans.main: Implementation of a Wilcoxon Signed Rank Test. **WilcoxonSignedRankTest(float[], float[])** - Constructor for class trans.main.WilcoxonSignedRankTest: Minimal constructor, fastest way to calculate a transformedPValueFloat **WilcoxonSignedRankTest()** - Constructor for class trans.main.WilcoxonSignedRankTest: **window(ExpressedGene[])** - Method in class expr.CorrelationMaps: Splits a chromosome of ExpressedGenes into windows based on the windowSize and minNumberGenes **Window** - Class in trans.main: Class to hold information about a window, a defined portion of the genome typically < 1kb. **Window(String, int, int, int, double[])** - Constructor for class trans.main.Window: **WINDOW\_SIZE** - Static variable in class edu.utah.seq.parsers.BarParser: **windowAverageScores(double[], int)** - Static method in class util.gen.Num: Slides a window along an array, one index at a time, averaging the contents. **windowAverageScores(float[], int)** - Static method in class util.gen.Num: Slides a window along an array, one index at a time, averaging the contents. **windowAverageScoresIgnoreScore(float[][], float, int)** - Static method in class util.gen.Num: Slides a window along an array, one index at a time, averaging the contents, ignores zero values. **windowAverageScoresIgnoreScore(float[], float, int)** - Static method in class util.gen.Num: Slides a window along an array, one index at a time, averaging the contents. **windowAverageScoresIgnoreZeros(double[], int)** - Static method in class util.gen.Num: Slides a window along an array, one index at a time, averaging the contents, ignores zero values. **WindowBlockMaker** - Class in trans.main: Converts a Window[] into a heat map specific sgr file for import into IGB. **WindowBlockMaker(int)** - Constructor for class trans.main.WindowBlockMaker: **WindowBlockMaker(int, String)** - Constructor for class trans.main.WindowBlockMaker: **WindowBlockMaker(String[])** - Constructor for class trans.main.WindowBlockMaker: **WindowBlockMakerTwoColor** - Class in trans.main: Converts a Window[] into a heat map specific bar file for import into IGB. **WindowBlockMakerTwoColor(int, String, String, double, double)** - Constructor for class trans.main.WindowBlockMakerTwoColor: **WindowBlockMakerTwoColor(String[])** - Constructor for class trans.main.WindowBlockMakerTwoColor: **WindowComparator** - Class in trans.main: Class to help sort an array of Window[] by chromosome, start position, length shortest first. **WindowComparator()** - Constructor for class trans.main.WindowComparator: **WindowHistogram** - Class in trans.misc: Makes a histogram of scores from a Window[]. **WindowHistogram()** - Constructor for class trans.misc.WindowHistogram: **WindowMaker** - Class in trans.tpmap: Creates Windows (start position, end position) of a given length, containing a given number of start positions and maximum number of features. **WindowMaker(int, int)** - Constructor for class trans.tpmap.WindowMaker: **WindowMaker(int, int, int)** - Constructor for class trans.tpmap.WindowMaker: **windowMidPoint(Window)** - Static method in class trans.misc.BestWindowScoreExtractor: **Windows2HeatMapSgr** - Class in igb.util: Converts a text file (columns: chrom, start, stop, score) into a heat/block map specific sgr file for import into IGB. **Windows2HeatMapSgr(String[])** - Constructor for class igb.util.Windows2HeatMapSgr: **windowScorePointData()** - Method in class edu.utah.seq.analysis.QCSeqs: Calculates window averages for merged PointData. **WindowScoreSmoother** - Class in util.apps: Slides a window across an array of numbers averaging the scores. **WindowScoreSmoother()** - Constructor for class util.apps.WindowScoreSmoother: **windowSum(int, int, int)** - Method in class edu.utah.seq.data.PointData: **winFileConvert(String, HttpServletRequest)** - Static method in class util.gen.IO: Checks to see if the browser sending the request is from a windows machine. **writeArrayList(ArrayList, File)** - Static method in class util.gen.IO: Writes each element of an Arraylist on a seperate line to a file. **writeBarFile(File, String, String, char, int[], float[], HashMap<String, String>)** - Method in class edu.utah.seq.parsers.BarParser: Writes a xxx.bar file to disk. **writeBinary(Coordinate[], File)** - Static method in class util.bio.annotation.Coordinate: Writes a binary Coordinate[]. **writeBinaryCoordinates(File, Region[])** - Static method in class trans.anno.Region: Writes the chrom start stop of a Region[] as a binary file. **writeBinaryInt2Array(int[][], File)** - Static method in class util.gen.Num: Writes a binary int[][2] array. **writeBinarySequence(String, File)** - Static method in class util.bio.seq.Seq: Writes a binary sequence, gatc, anything else is assumed to be n. **writeBindingRegions(BindingRegion[], File)** - Static method in class trans.anno.IntersectRegions: **writeCelaFiles()** - Method in class trans.misc.ConvertAgilentData: **writeCelFile()** - Method in class trans.graphics.CelMasker: **writeConsensusBars(String, float[])** - Method in class edu.utah.seq.parsers.ElandSequenceParser: **writeDocumentToOutput(Node, int)** - Method in class util.xml.ParseXMLFile: Writes node and all child nodes into System.out **writeEncryptedCredentialsFile(File, String, String, String)** - Static method in class util.amazon.S3Manager: Write a password protected encripted credentials file. **writeEnrichedRegions(File)** - Method in class edu.utah.seq.analysis.EnrichedRegionMaker: Writes out ER reports. **writeGATCBars(String, int[][])** - Method in class edu.utah.seq.parsers.ElandSequenceParser: **writeGenesToOutput(Node)** - Method in class util.xml.ParseXMLFile: Writes node and all child nodes into System.out **writeGrFile(File)** - Method in class trans.misc.GrGraph: Writes to the file base tab value. **writeHashMap(HashMap, File)** - Static method in class util.gen.IO: Writes each key tab value on a separate line to the file. **writePNGs(boolean)** - Method in class trans.qc.CelFileQualityControl: Writes PNG files for CelFileStats. **writePointData(File)** - Method in class edu.utah.seq.data.PointData: Writes a zip compressed 'chromosome\_strand\_.bar.zip' file into the saveDirectory. **writePointData(HashMap<String, PointData>, File)** - Static method in class edu.utah.seq.data.PointData: Writes zip compressed 'chromosome\_strand\_.bar.zip' files into the saveDirectory. **writePointDataToFile(File)** - Method in class edu.utah.seq.data.PointData: Writes a zip compressed 'chromosome\_strand\_.bar.zip' file. **writeReport()** - Method in class selex.SelexParams: **writeRScriptFile()** - Method in class util.bio.wrappers.Segment: **writeSGRFile(RocWindow[], File)** - Static method in class trans.roc.ParsePatternedWindows: Writes an sgr file. **writeSgrFiles()** - Method in class trans.misc.ConvertAgilentData: **writeSimpleBarFile()** - Method in class edu.utah.seq.parsers.BarParser: Writes a simple bar format graph using the BarParser field values. **writeSimpleBarFile(GrGraph, File)** - Static method in class trans.misc.Util: Writes a simple bar format graph for a xxx.gr type graph. **writeSimpleBarFile(String, String, String, int[], float[], File)** - Static method in class trans.misc.Util: Writes a simple bar format graph for a xxx.gr type graph. **writeString(String, String)** - Static method in class gata.main.GATAUtil: **writeString(String, File)** - Static method in class util.gen.IO: Writes a String to disk. **writeString(String, String)** - Static method in class util.gen.IO: Writes a String to disk. **writeStringAppend(String, File)** - Static method in class util.gen.IO: Writes a String onto the end of a file. **writeSubSeqFasta()** - Method in class selex.SelexParams: **writeSubWindowBarFiles(File, String)** - Method in class edu.utah.seq.analysis.EnrichedRegionMaker: Writes the sub windows for each enriched region, note also sorts enriched regions by position **writeTableOfIntensities()** - Method in class trans.misc.CalculateGeneExtensions: **writeTagValuePairs(DataOutputStream, HashMap<String, String>)** - Static method in class edu.utah.seq.parsers.BarParser: **writeTempFile(Object, HttpServletRequest, String)** - Static method in class bioroot.antibody.AntibodyBase: Looks for an uploaded file, writes it to the directory with a tempRNDMWord\_FileName, will return the name, or null, or 'Too big' **writeToFile(float[], File)** - Static method in class util.gen.Num: Writes a float array to file, one per line. **writeToFile(double[], File)** - Static method in class util.gen.Num: Writes a float array to file, one per line. **writeTxtRocFile(RocWindow[], File)** - Static method in class trans.roc.ParsePatternedWindows: Writes a chrom, start, stop, score file.

---


|  |  |  |
| --- | --- | --- |
| **Overview** \| Package \| Class \| Use \| **Tree** \| **Deprecated** \| **Index** \| **Help** | |  |
| **PREV LETTER**   **NEXT LETTER** | **FRAMES**    **NO FRAMES**     **All Classes** |  |


A B C D E F G H I J L M N O P Q R S T U V W X Z 

---
